# Detecting Leadership Opportunities in Group Discussions Using Off-the-Shelf VR Headsets

**DOI:** 10.3390/s24082534

**Published:** 2024-04-15

**Authors:** Chenghao Gu, Jiadong Chen, Jiayi Zhang, Tianyuan Yang, Zhankun Liu, Shin’ichi Konomi

**Affiliations:** 1 Graduate School of Information Science and Electrical Engineering, Kyushu University, Fukuoka 819-0395, Japan; 2 Faculty of Arts and Science, Kyushu University, Fukuoka 819-0395, Japan

**Keywords:** virtual reality, group work, human–computer interaction, sensor, leadership, deep learning

## Abstract

The absence of some forms of non-verbal communication in virtual reality (VR) can make VR-based group discussions difficult even when a leader is assigned to each group to facilitate discussions. In this paper, we discuss if the sensor data from off-the-shelf VR devices can be used to detect opportunities for facilitating engaging discussions and support leaders in VR-based group discussions. To this end, we focus on the detection of suppressed speaking intention in VR-based group discussions by using personalized and general models. Our extensive analysis of experimental data reveals some factors that should be considered to enable effective feedback to leaders. In particular, our results show the benefits of combining the sensor data from leaders and low-engagement participants, and the usefulness of specific HMD sensor features.

## 1. Introduction

Advancements in consumer-level head-mounted displays and computer graphics are paving the way for virtual reality to enhance online and remote learning closer to real-world settings [1]. The absence of some forms of nonverbal communication in VR, however, can make communication and interaction more difficult compared to face-to-face settings [2]. Although nonverbal communication such as para-language, facial expressions, and posture plays a vital role in human interaction and communication [3,4,5], it is hard for people to interpret the expression and specific body motion of other group members accurately when using avatars in virtual reality [2].

Studies have emphasized the importance of leaders in virtual teams [6,7,8]. Leadership has been shown to directly affect the dynamics, work standards, and communication protocols of virtual teams [8]. In particular, distributed teams can perform better when leaders create pressure and awareness of others’ contributions [7]. This suggests the importance of supporting a leader’s awareness of other group members, which could assist the leader in organizing discussions more effectively. Moreover, due to the complexity of leadership, not everyone possesses the same capability for leading group discussions [9]. People use different styles of leadership, sometimes failing to lead group discussions or teamwork efficiently. For instance, autocratic-style leaders assert social dominance by monopolizing a significant portion of the speaking opportunities, resulting in limited chances for other members to participate and engage in discussions [10], while laissez-faire style leaders may lack confidence to facilitate efficient discussions, thereby causing protracted decision-making processes [10]. In virtual environments, communication between leaders and group members may be subject to some obstacles. In this context, there are opportunities to support leadership for the success of group discussions. For example, the difficulty of exchanging some non-verbal cues in VR may hinder leaders from being aware of group members with suppressed speaking intentions. Leaders relying solely on avatars may fail to understand members’ emotions and attention, which could impede their efforts to facilitate discussions efficiently. In particular, the lack of some non-verbal cues may prevent leaders from supporting turn-taking behaviors effectively during discussions. The sensor data available on off-the-shelf VR devices could provide a wealth of non-verbal information related to head movements, gestures, body movements, and so on, and they might potentially help alleviate the difficulty of communication in VR group discussion. Both verbal cues and non-verbal cues such as prosodic means, gaze, head movements, gestures, and body posture play essential roles [11,12,13] for leaders to understand the need and timing for proper interventions. We posit that motion data, including head movement, body movement, and hand gestures data, could be used to support leaders with the awareness of leadership opportunities and minimize problematic situations in VR-based group discussions such as the ones caused by suppressed speaking intention.

In this paper, our main objective is to detect opportunities for facilitating engaging discussions and support leaders in group discussions using off-the-shelf VR headsets. In particular, we propose a novel detection approach that exploits social behavior patterns in leader-led group discussions to provide awareness about problematic situations caused by suppressed speaking intentions. Based on the sensor data collected during the VR group discussions experiment used to detect suppressed speaking intentions, we employ group members’ sensor features to construct personalized and general deep learning models for detecting suppressed speaking intentions. Personalized models assist us in understanding the model’s detection capabilities on similar but unseen data, and general models help us to better assess the model’s detection capabilities on data from new users. We posit that the existence of leaders can influence the behavior of group members during group discussions and simultaneously consider the sensor features of a leader and other group members to detect suppressed speaking intentions. In this context, our research questions are as follows. RQ1: Can we detect opportunities for facilitating engaging VR group discussions based on the sensor data from commodity VR devices by using personalized and general models? RQ2: Do leaders’ sensor features contribute to the detection of members’ suppressed intentions to speak? RQ3: Which type of leaders’ sensor features contribute the most to the detection?

Our main contributions are as follows.

1. MODELS: We propose general and personalized models following architectures of three commonly used time-series classification models for detecting leadership opportunities focusing on suppressed speaking intentions. Our model only requires the collected data from off-the-shelf VR devices without the need to use additional sensing devices.
2. FEATURES: We show that our personalized models can improve the detection performance by integrating the sensor data from group leaders and members. We also uncover the usefulness of the leaders’ sensor data in detecting the speaking intentions of low-engagement group members.
3. SENSOR TYPES: Furthermore, we investigated which type of leader’s sensor features, including HMD, controllers, position, and rotation in a virtual environment, contribute the most to the performance improvement. The results suggest that HMD sensor features contribute the most to the performance improvement of personalized models for the majority of low-engagement members.
4. IN-DEPTH ANALYSIS: We analyze the behavior patterns in the VR group discussion through video analysis and discuss the reasons why leaders’ sensor data can improve the detection performance for low-engagement group members. We then discuss their implications for the design of support mechanisms in VR group-discussion environments.

## 2. Related Works

Existing research in supporting group discussions has revealed some issues regarding group formation, group dynamics, and the learning process. These issues can be traced back to impeded social interactions between group members [14]. To address social issues in face-to-face group discussions, researchers have explored various strategies. For instance, Dagan et al. developed a social wearable prototype aimed at enhancing individuals’ awareness of and control over their verbal participation in group discussions [15]. The device they proposed detects the wearer’s speech patterns and provides visual and tactile feedback as needed. The OpenMic utilizes proxemic metaphors in videoconferencing to manage conversational dynamics, employing features like the Virtual Floor and Malleable Mirrors to facilitate smoother interactions in multi-party video meetings [16]. Additionally, some studies have leveraged group awareness tools to improve communication and collaboration within groups [17,18,19]. Other research works shared sensor data (e.g., location and activity stream) and biosignals (e.g., heart rate) to facilitate social interaction [20,21,22].

Non-verbal features such as head movement, gaze, and hand gestures, as well as interaction features, have been commonly employed to analyze social and group dynamics. For instance, Chen et al. introduced a Kinect-based method for evaluating group discussion behavior by utilizing algorithms to detect each participant’s facial direction and infer their roles within the discussion [23]. Muller et al. extensively analyzed non-verbal signals to detect low rapport in small groups and explored the automated prediction of low rapport during natural interactions in these group settings [24]. Furthermore, biosignals and sensors from commodities’ VR devices have also been utilized in some recent studies. In recent years, Zhang et al. have shown that the behavior features extracted from the wearable sociometric badge sensor provide useful information in assessing personal affect and team cohesion [25]. Chen et al. have investigated the use of sensor data from off-the-shelf VR devices, along with additional contextual information such as user activity and engagement, to predict opportune moments for sending notifications using deep learning models when users interact with VR applications [26]. Tan et al. propose an LSTM-based head orientation estimation method with alternative non-verbal inputs, like speaking status, body location, orientation, and acceleration [27]. Despite the many efforts to enable the sensor-based detection of non-verbal features, none of them fully consider the characteristics of leader-led group discussion to detect and support leadership in VR-based groups.

Leadership is a critical factor in both real-world groups and virtual teams during discussions and collaborative work [8,10,28]. Everyone possesses different capacities for leading teams effectively, given the complexity of leadership [9]. Consequently, studies have been conducted to explore tools to support leadership [29,30] and understand the dynamics of leadership roles within group discussions [31]. In addition, leaders employ various methods for applying leadership, such as one-on-one feedback sessions, speeches, and non-verbal behavior [30]. Some works utilized graph link analysis and social network analysis to analyze and detect leadership within groups [31,32].

Most of these previous studies focus on face-to-face scenarios and demand substantial expertise and effort to construct features before employing machine learning models. Again, our work focuses on an important yet relatively unexplored setting of groups with a leader in VR and proposes techniques that suit such a setting. It is worth noting that an earlier paper introduced a practical system designed to detect a user’s intent to speak to a computer [33]. However, this work primarily centered around audio, facial expression, and facial feature analysis, and it primarily addressed scenarios where users were talking to computers. This distinguishes it from our approach to detecting speaking intention. Unlike our previous work [34], which centered on free discussions without leaders, our current research focuses on leader-led VR group discussions in the context of a survival game scenario and proposes novel techniques for the detection of suppressed speaking intention. We have also modified our data sampling approach, adopting a more general slide window method instead of solely sampling data from the first 3 s before speaking. Additionally, our current work includes the consideration of the unachieved speaking intention labels and simultaneously trains personalized and general models on the dataset of group members.

## 3. Experiment Design

We conducted an experiment to understand experiences and behaviors in VR-based group discussions involving a leader and to explore techniques to detect leadership opportunities focusing on suppressed speaking intentions. In this section, we describe the design of the experiment, including the experimental setup and procedure.

### 3.1. Participants

We recruited 24 participants (12 females, 12 males), who are university graduate students aged 22–30 (M = 25.5, SD = 2.27). All participants were asked about their VR experiences before the experiment. Thirteen participants (54%) have prior VR experiences, and 11 participants (46%) have no prior VR experiences. As for the participants without prior VR experience, we provided VR devices for them to familiarize themselves with the basic operations of the VR equipment prior to the experiment, thereby minimizing the novelty effect. We also asked whether participants experienced severe 3D motion sickness and discomfort during the use of VR. All participants responded that they experienced neither 3D motion sickness nor discomfort in VR environment.

### 3.2. Experimental Setup

**Figure 1:**
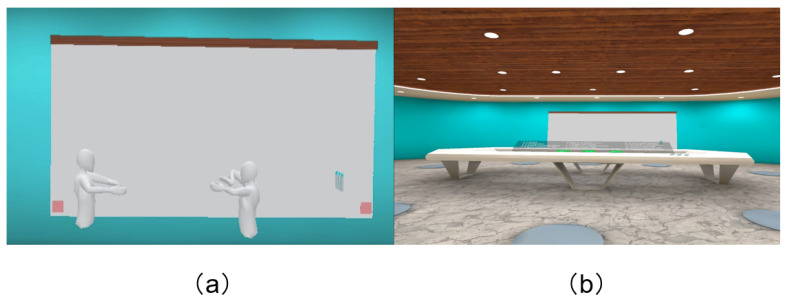
The virtual environment of group discussion (**a**). Avatars in the virtual environment (**b**).

The experimental virtual environment (VE) we developed used the Unity 3D game engine’s XR interaction tool to collect participants’ motion sensor data. The VE resembles a meeting room and includes a large table and two whiteboards (see Figure 1b). Participants can manipulate their avatars to move freely, write on the whiteboard with a pen, and communicate with others in the VE. We used the consumer-level VR device Oculus Quest2, which consists of two controllers and an HMD. During the group discussion, participants can utilize the whiteboard to write their ideas and engage in discussions with others. Avatars in the VE are relatively simple and monochromatic. Since our study primarily focuses on using motion sensor data available on off-the-shelf VR devices and studying the relationship between motion data and speaking intention, the avatars do not have facial expressions (see Figure 1a).

Participants were divided into groups of four, and each group entered the virtual environment for VR discussion scenario involving a leader. Even though the group discussion activity being simulated in this experimental setup typically involves 4–5 participants per group, we opted for a 4-person group in this experiment to minimize complexity. During the experiment, each group of participants would sit together in a room, with a certain distance maintained between each individual within the group. Participants wear the head-mounted display (HMD) and use hand controllers to interact with the objects in the virtual environment.

### 3.3. Procedure

The experiment was conducted in groups. In order to ensure gender balance and mitigate the influence of gender on the experiment, 24 individuals were randomly assigned to six experimental groups, with four participants (two males, two females) in each group. In each group, participants are acquainted with each other, though not extensively familiar. Firstly, the experimenter provided them with a comprehensive overview of the study and its protocol. Subsequently, we presented a demonstration video and provided instructions on accessing the experimental virtual environment (VE), followed by an explanation of the VR discussion scenario. Before entering the experimental virtual environment, participants engaged in a practice session within the VE to familiarize themselves with the basic operations of VR devices. The duration of all the aforementioned sessions is 20 min. Figure 1b shows the virtual environment for our experiment.

Prior to the onset of the formal experiment, four participants would greet each other and familiarize themselves with the fundamental functionalities (e.g., movement and using the whiteboards) in the virtual environment within 5 min. We also would explain the instructions given to the participants about the leader and what a leader should do in the discussion. Then, they would engage in a 5-min communication to select a leader who would provide guiding advice, organize communication, and be engaged in group decision-making during discussion. In addition, all participants were instructed to record videos from their first-person perspective from the beginning of the discussion for later annotation and analysis. During this scenario, a group of four participants is engaged in a survival game known as “Lost at Sea” [35]. The task of the group is to rank 15 items based on their perceived importance for survival. Following a 20-min discussion, the group is required to provide a final ranked list of the 15 items on the virtual environment’s whiteboard. This scenario is characterized by problem-based communication. This survival game provides the group with a clear discussion objective and, to some extent, enhances interpersonal communication among participants. Moreover, throughout this process, the leader can comparatively easily demonstrate their leadership, facilitating our analysis of the impact of the leader’s actions on the speaking intentions of group members. Upon completion of the scenario and after the subsequent 5-min break, participants were instructed to review the recorded videos and annotate the time intervals during which they had the intention to speak within 35 min. Each participant was involved solely in their annotation process. Finally, participants were requested to complete a questionnaire in 10 min regarding their VR discussion experience.

**Figure 2:**
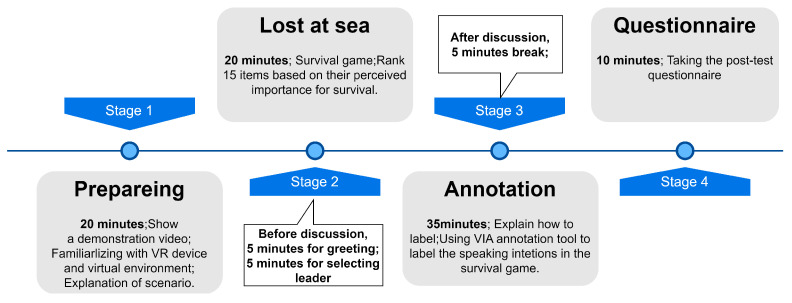
Procedure of VR-based group discussion experiment.

Before starting the discussion, participants were instructed to utilize the built-in screen recording function of the VR devices to capture video footage from a first-person perspective. This recorded video material is intended for retrospective review and annotation purposes. The experiment lasted for a total of 1 h and 40 min. Figure 2 shows the whole process of our VR group discussion experiment.

We took into account these two types of intentions to speak: achieved speaking intention and unachieved speaking intention. Regarding achieved speaking intentions, based on the group’s turn-taking mechanism [36,37], if listeners have the intention to speak, the listeners may proactively attempt to take the floor; if speakers still harbor the intention to speak after the previous speech, they may continue to keep the floor. For unachieved speaking intentions, group members may, due to various reasons, hold the intention to speak during a certain period but ultimately refrain from vocalizing it. Before participants apply labels, we have provided some examples of speaking intentions to help participants understand the concept of ‘intention to speak’. Here, are a few examples of the intention to speak in group discussion scenarios.

1. When there is no ongoing conversation and you intend to initiate a discussion on a new topic.
2. When you intend to participate in a discussion among other individuals on a topic.
3. When you intend to express your own thoughts or provide a response in a discussion on a topic.

After the annotation process, 24 participants (18 group members, 6 leaders) were required to fill out a questionnaire investigating the questions presented in Table 1. The reason for including Q1 in our survey was to obtain a better understanding of the reason for unachieved speaking intentions and to provide some insights into how the intentions to speak of members could be utilized to assist leaders in facilitating group discussions. In our survival game, we aim for the leader to actively participate in the discussion and guide the group members’ deliberations. However, the process of group decision-making involves discussions and decisions made collectively by all members within the group. This represents a form of democratic leadership (participatory leadership) [10,38,39]. Thus, Q2 and Q3 assist us in gaining a more comprehensive understanding of the contributions in group discussions and the influence of leaders on group discussions. For open-ended questions Q1 and Q3, the researchers engaged in comprehensive discussion about and coding of each participant’s responses and then categorized the coding results.

## 4. Data Collection

In the virtual meeting room we constructed, we utilized VR devices to collect participants’ positions, movements, and other physical actions during the discussions. The VR device employed in our experiment, Oculus Quest 2, is capable of providing sensor data from three components: the head-mounted display, the left-hand controller, and the right-hand controller. Each component enables data collection pertaining to position, rotation, velocity, acceleration, angular velocity, and angular acceleration.

Unlike group discussions in the physical space, participants in VE can utilize the VR controllers to move and rotate the avatar, grab the pen, and write on the whiteboard in the virtual environment. Therefore, we additionally collect virtual sensor features that represent the position and rotation of the user in the virtual environment respectively. The experimental application we developed is capable of uploading sensor data to the database at a stable frequency of 20 Hz.

For our annotation strategy, all participants are first instructed to use the video annotation tool called VGG video annotator [40,41] to review the recorded videos and mark the start point of each speech. Then, they are required to determine whether the speech is an instance of active speaking. If it is an instance of active speaking, participants need to mark the time interval starting from when they had the intention to speak (with the start of the intention as the starting point) to when they started speaking as the endpoint. Additionally, participants were asked to mark the time intervals for instances where they held the intention to speak but did not speak out during the video review process.

**Figure 3:**
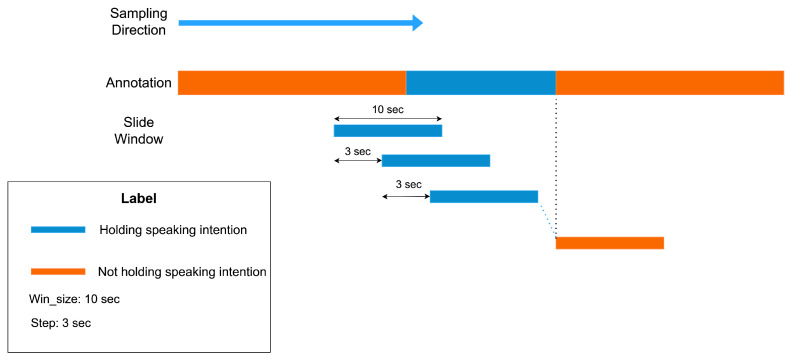
Sample data points from continuous annotated data using sliding window method.

We collected sensor data that occur at a frequency of 20 Hz. According to our research questions and purpose, we utilized the sensor data and labels from the 18 group members to construct the training and testing datasets, with data from the six leaders employed as contextual information for investigating RQ2. A total of 380,918 sensor data entries were collected from 18 group members during the 20-min survival game discussion. Among these, only 47,262 (≈12.4%) data entries were identified as positive samples (with the intention to speak), while the remaining data points were negative samples. Hence, it is evident that there is a significant class imbalance between positive and negative samples. The annotation tool and our annotation strategy gave us continuously labeled data. As a result, we used a sliding window to sample data points. The sensor data points in each time window are used for the detection of speaking intentions during that period of time window. We used ten seconds as window size and three seconds as step size because it resulted in the best performance among five candidate window sizes (2 s, 3 s, 5 s, 10 s, and 15 s) and four candidate step sizes (0.5 s, 1 s, 3 s, and 5 s) in our detection results. Figure 3 shows the process of sampling data points by applying the slide window approach. Additionally, a slightly larger window can alleviate the overall imbalance between positive and negative samples; if the window size is too small, it would increase the difficulty of the model in recalling positive samples.

**Figure 4:**
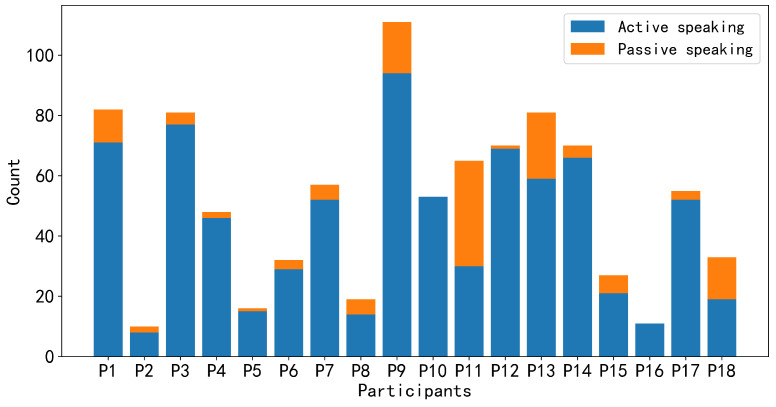
The number of utterances made by each group member during the discussion.

**Figure 5:**
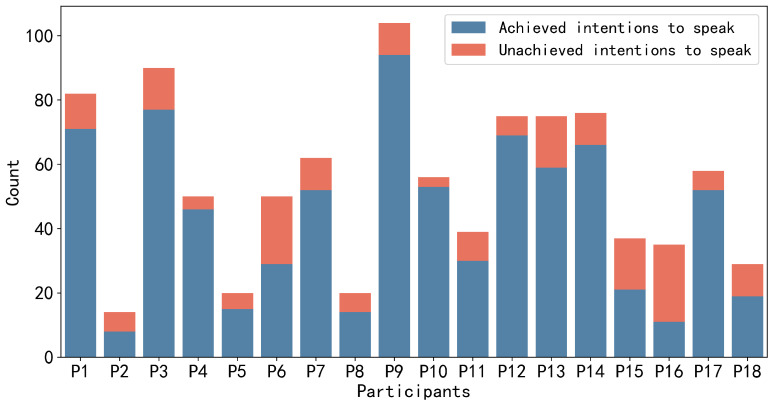
The number of speaking intentions labels annotated by each group member during the discussion.

For the annotation data, there are a total of 972 instances of speaking intention, with 786 (≈81%) annotated as achieved and 186 (≈19%) annotated as unachieved. The total duration of speaking intention labels for the 18 participants was 1232 s (≈20 min). Each group member annotates different frequencies and durations of speaking intentions. Participants with higher engagement in the discussions have a larger number of speaking intentions than others. However, in terms of those with lower engagement, not only did they have fewer speaking intentions but they also spoke less frequently during the discussions. Figure 4 and Figure 5 depict the number of utterances made by each group member during the discussion and the frequency of speaking intentions possessed by each group member during the discussion, respectively. In Figure 4, based on our annotation strategy, active speaking indicates that participants want to actively express their own views or respond to others’ opinions voluntarily. Passive speaking indicates that participants may be influenced by the will of others and are compelled to express their views, such as being called upon by other members to present their opinions. Since we consider speaking intentions to be more inclined towards participants actively expressing their views, we instructed participants to focus on active speaking and unachieved speaking intentions during the labeling process. It is worth noting that even after applying the sampling methods described above, an imbalance still exists between positive and negative samples in the data of some group members. For instance, after employing sliding window sampling, P2, P5, P8, P18, P15, and P16 have positive samples accounting for only around 13.5%, 16.4%, 18.6%, 24.1%, 31.6%, and 35.1%, respectively.

In sampling the data points, we ignored data points that would contain noise, which were points that satisfied either of the following conditions. First, we excluded certain data points from the initial and final stages of the experiments. During the initial stages of the experiments, some participants may exhibit interactions with the virtual environment that are less familiar, resulting in discussions and actions unrelated to the experiment. Similarly, during the final stages of the experiments, due to the fact that some groups finished the discussion prematurely, participants engaged in activities unrelated to the experiment. For instance, some participants set aside their VR controllers and took a break. Second, we removed data points associated with the period where participants inadvertently moved outside the VR movement boundaries or experienced unexpected incidents such as unintentional button presses on the HMD resulting in a black screen interface during the discussion. Finally, participants are not consistently precise when annotating the starting point of speech (the endpoint of achieved speaking intentions labels). We employed the Whisper [42] tool from Open AI to assist in correcting the endpoints of some labels with large deviations. This sampling method produced a dataset containing 5717 total samples, consisting of 2600 positive samples (with intention to speak) and 3117 negative samples (without intention to speak).

Finally, we describe the data collected through the questionnaire survey. Firstly, regarding Q1, the responses from the 24 participants varied. Five participants indicated that although such situations occurred, they did not know the reason (20.8%). Four participants mentioned that they did not want to interrupt other members’ discussions at that moment (16.7%). Another four participants refrained from speaking because what they intended to say was unrelated to the ongoing discussion (16.7%). Additionally, four participants felt that they were not sure how to express their thoughts at that time (16.7%). Three participants thought that other members had already expressed similar ideas, so they decided not to speak (12.5%). Moreover, two participants mentioned missing the timing for contributing to the discussion (8.3%), and another two were concerned about speaking too much (8.3%). One participant stated that they had unique questions about some viewpoints but were worried that other members would not understand their ideas (4.2%). Another participant mentioned not wanting to oppose others’ opinions (4.2%). Finally, one participant felt that the discussion was coming to an end, so they chose not to speak (4.2%). In summary, the most common reason was not wanting to interrupt other members’ discussions.

**Figure 6:**
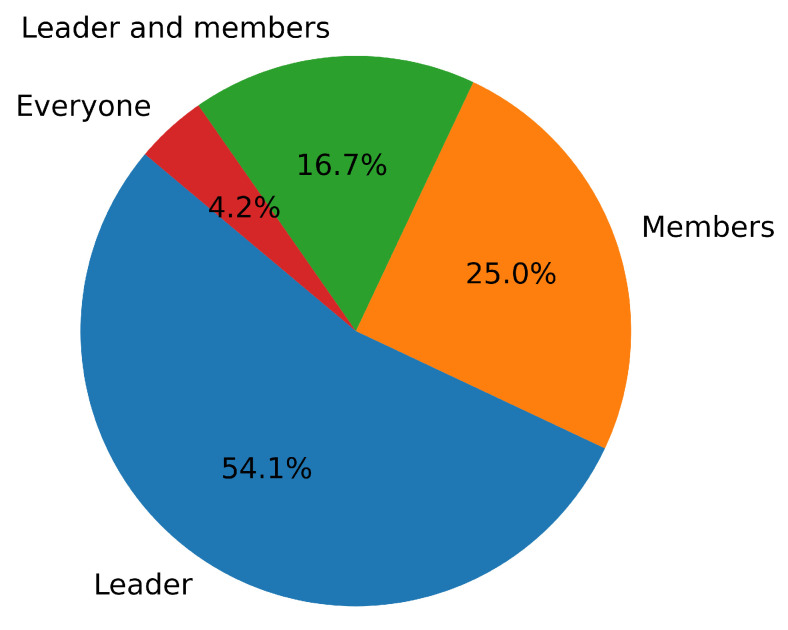
The overall results of who participants think contributed the most to the discussion.

Secondly, Q2 was measured via the five-point Likert scale, with 1 = “strongly disagree” and 5 = “strongly agree”. The overall satisfaction level of the 24 participants with the leader was relatively high (M = 4.45, SD = 0.66), with leaders reporting lower self-satisfaction (M = 3.67, SD = 0.56) compared to the satisfaction of members with leaders (M = 4.72, SD = 0.42). In terms of Q3, Figure 6 shows the overall results of who participants think contributed the most to the discussion. Overall, most participants (54.1%) considered the leader to be the most important contributor in the group discussions. Some participants (25%) also believed that some group members actively expressed their opinions and made more contributions during the discussions. Additionally, 16.7% of participants thought that both the leader and some members actively participated in the discussions and made significant contributions. One participant (4.2%) believed that everyone in the group contributed equally. Among the results from the six leaders, four of them believed that they had the highest contribution, while two leaders thought that some members contributed more within the group. The total survey results can provide supplementary explanations to enhance our understanding of leadership and member participation within the group.

The data we collect in VR environments, including motion sensor data, video data, and questionnaires from participants, may contain personally sensitive information. Systems that exploit such data to facilitate group discussion should respect users’ privacy and autonomy through user-centered design processes that mitigate the users’ risks and concerns. In our experiment, we utilized anonymous identifies when collecting and managing experimental data. Before collecting user data, we obtained informed consent from the participants, who clearly understood the purposes, scope, and potential risks associated with the collection, use, and sharing of their sensor data. Furthermore, participants’ data are stored on our lab server with access controls in place.

## 5. Analysis and Result

### 5.1. Deep Learning Model

**Figure 7:**
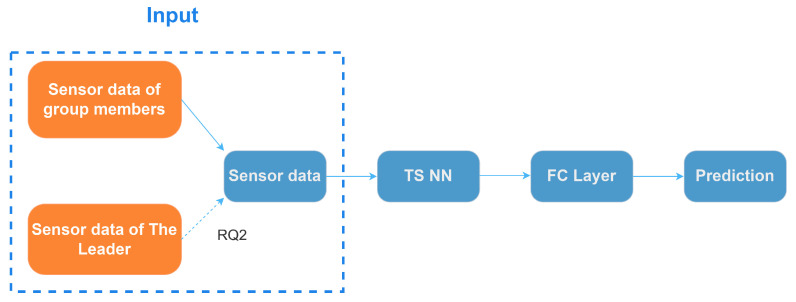
The pipeline of our NN-based method. Legend: “TS NN”: Time-Series Neural Network, “FC Layer”: Fully Connected Layer. The dashed line signifies the integration of leader sensor features for addressing RQ2.

To examine the feasibility of the sensor-based detection of leadership opportunities in VR group discussions (RQ1), we attempt to predict group members’ suppressed intentions to speak during VR-based group discussion using time-series sensor data, which is a typical time-series classification task [43]. Neural network (NN)-based models (such as the Convolutional Neural Network [44] and the Recurrent Neural Network [45]) have achieved success in time-series classification tasks. Thus, we also used NN-based models to process our time-series motion sensor data. To find and examine which time-series classification methods worked the best in detecting suppressed speaking intention in VR-based group discussion, we explored three commonly used time-series classification models, including a one-dimensional convolutional neural network (1D-CNN) with a small and compact architecture (EEGNet [46]); an ensemble of deep Convolutional Neural Network models inspired by the Inception-v4 [47] architecture (InceptionTime [48]); and multivariate long short-term memory fully convolutional network (MLSTM-FCN [49]), which combines the use of 1D-CNN and long short-term memory (LSTM). The pipeline of our approach is shown in Figure 7. To examine the feasibility of detection (RQ1), the input sensor data comprises data from 18 group members. To analyze the impact of leaders’ sensor features (RQ2), the input sensor data are combined with the sensor features of each group member’s corresponding leader in timeline order, forming the input sensor dataset.

We implemented the NN-based models using keras [50], a widely used deep-learning framework for Python. We trained these models end-to-end using binary cross-entropy loss and an Adam optimizer with an initial learning rate of $10^{-1}$ and a batch size of 64. The learning rate was decreased over time to reach $10^{-4}$. Moreover, we used the network architecture of these three models and only modified the layers’ configuration to better fit our dataset. Table 2 shows the layer details of the best-performing EEGNet. In Table 2, *C* denotes the dimensionality of features during model training. For both the general model and the personalized model, the value of *C* is 60, indicating that our input data samples possess 60 features. Among these, the number of HMD features is 18, the number of features for the left and right controllers is 36, and the number of features representing the position and rotation in the virtual environment is 6. In RQ2, after concatenating the leader’s sensor features along the time axis, the value of *C* becomes 120.

### 5.2. Validation Method

We use the aforementioned time-series classification models to train personalized and general models and compare their performance using different metrics. This also allows us to investigate whether personalized or general models perform better in predicting group members’ suppressed intentions to speak. Initially, we trained personalized models for each group member. We partitioned each group member’s data into training and testing sets based on time intervals while preserving the temporal continuity of the data within those intervals. For example, in a 20-min survival game, we used 18 min of data for training and the remaining 2 min for testing. Thus, similar to some studies [26,51] involving predictive work using sensor data, we employed 10-fold cross-validation for each group member, with 10% of the data designated as the test set and 90% as the training set. However, as indicated in Figure 4 and Figure 5, the number of speaking intentions varied for each individual. Some participants with low engagement or less proficiency in communication had a limited number of speaking intentions, meaning that the quantity of positive samples was significantly lower than that of negative samples. As previously mentioned, even after sampling the data using a sliding window approach, an imbalance still exists between positive and negative samples in the data of some group members. Therefore, when training personalized models for each group member, we omitted runs in which the entire test set consisted of negative samples or rounds with only negative or positive samples in the training set. After excluding these extreme rounds, the originally 180 runs of training were reduced to 166 runs. The result is obtained by averaging the results across all runs for each group member individually and then averaging the results across all 18 group members. Personalized models assist us in understanding the model’s detection capabilities on similar but unseen data.

To explore the generalization capability of sensor features across different group members, we similarly employed a leave-one-member-out cross validation [24,26] to build a general model. This approach involves selecting one individual as the test set in each run, with the remaining 17 individuals serving as the training set. The final result is the average of the results from 18 runs. Building a general model helps one to better assess the model’s detection capabilities on data from new users. Regarding model evaluation metrics, we employed accuracy, F1-score, precision, recall, and AUROC to comprehensively assess the model’s performance. Accuracy is the most commonly used evaluation metric, measuring the proportion of correct predictions. However, in our study we aimed to achieve high precision and recall of positive samples during group discussions to provide more accurate and useful insights to the leader. Therefore, only considering accuracy does not meet our requirements. Hence, we focused more on the other evaluation metrics. Precision indicates the proportion of correctly predicted speaking intention samples among all predicted speaking intention samples. Recall represents the proportion of correctly predicted speaking intention samples among all true speaking intention samples. F1-score is the harmonic mean of precision and recall, providing a balance measure between precision and recall. In general, the higher the F1-score value, the better the model’s performance is considered. We also utilized the AUROC, which is the area under the receiver operating characteristic curve to assess the model’s overall performance, where a higher AUROC value indicates better model performance.

### 5.3. Analysis of Model Performance

#### 5.3.1. Performance of Personalized and General Models

For our RQ1, we examined the performance of predicting using sensor data through two approaches: personalized and general models. Our baseline model is a random classifier, which assigns data as positive or negative samples with an equal 50% probability. Table 3 shows the performances of personalized and general models. Overall, our results indicate that personalized models outperform general models across various evaluation metrics. For the general models, EEGNet exhibited superior overall performance compared to other models, with the highest Accuracy (0.5823), AUROC (0.5633), and Precision (0.5041). Moreover, all general models demonstrated better performance than the baseline. On the other hand, each personalized model’s performance across various evaluation metrics not only significantly surpasses the baseline performance but also outperforms all general models. Among the personalized models, EEGNet performs the best, with the highest Accuracy (0.6813), F1-score (0.7132), Precision (0.6201), Recall (0.8393), and AUROC (0.6058). In conclusion, the total results suggest that motion sensor features can assist in predicting speaking intentions in VR-based group discussions.

#### 5.3.2. The Leader’s Contextual Influence on Members’ Speaking Intention Prediction

To investigate whether the leader’s sensor data has an impact on predicting the speaking intentions of group members, we concatenated the leader’s sensor data as contextual information with the member’s sensor data along the time axis. According to the results and Figure 4 and Figure 5, we found that for those members who spoke infrequently and had fewer achieved speaking intentions during the discussion (low-engagement), leader’s sensor features improved their personalized models’ ability to detect speaking intentions. Furthermore, the leader’s sensor features were unable to significantly enhance the F1-score of personalized models for those with relatively high engagement levels. For the vast majority of those group members on or slightly below the medium level engagement, Leader’s sensor features were able to moderately improve the F1-score. Table 4 and Table 5 present the comparative results for the personalized models (EEGNet, MLSTM-FCN) of those members who were not actively involved in the discussions (P2, P5, P8, and P16).

It is worth mentioning that, in the above results, considering that our experimental scenario is task-based group discussions, we primarily evaluated the level of group members’ engagement based on their behavioral engagement [52,53]. We not only considered the frequency of their utterances but also reviewed the group discussion videos to observe group members’ other behaviors that contribute to the discussion. Based on our video observations, we found that P16 rarely initiated his own opinions during the group discussions and did not participate in the ranking activity on the whiteboard in VE. Even when he expressed opinions, it was mostly to complement the leader’s or other members’ ideas. P5 occasionally expressed her opinions during the group discussions but spent most of her time listening to others’ viewpoints and looking at others’ ranking activity on the whiteboard. The behaviors of P8 were similar to those of P16 and P5. P2 had the lowest frequency of speaking and the lowest number of speaking intentions among all group members. During the discussion, P2 participated in sorting two survival items on the whiteboard under the leader’s guidance but spent the remaining time listening to and observing the discussions and ranking activities of other group members. Additionally, other members (P6, P15, and P18), although they exhibited moderate frequency of speaking, actively engaged in the ranking activity on the whiteboard during the whole group discussion. We regarded P2, P5, P8, and P16 as low-engagement group members. Since the overall results of EEGNet are the best among all personalized models, we mainly discuss the results of EEGNet.

According to Table 4, the personalized model for P2 showed a significant improvement in predictive performance after incorporating the leader’s sensor features, with accuracy increasing to 0.6738, F1-score increasing to 0.7354, Precision increasing to 0.7213, and AUROC increasing to 0.5442. While not all of the evaluation metrics improved for these less actively engaged members, all of them showed improvements in F1-score and Precision, indicating that using the leader’s sensor data as context information can enhance the personalized model’s ability to accurately predict and recall positive samples. Additionally, it should be noted that the leader’s sensor data did not lead to significant improvements for those group members who have relatively higher engagement during group discussions, like P1, P3, P7, P9, P12, P13, P14, and P17; in some cases, it even resulted in an obvious decline in predictive performance. For example, the context personalized model for P1, P3, P9, P13, and P17, respectively, exhibited a 7.9%, 11.3%, 13%, 11.6%, and 13.5% decrease in F1-score. For P7, P12, and P14, with the relative improvement in F1-score being quite modest (less than 2%). Among those group members on or slightly below the medium level engagement (P4, P6, P10, P11, P15, and P18), P4, P6, P10, and P11 demonstrated respective increases of 7.9%, 8.1%, 3.2%, and 6.3% in their F1-scores. Meanwhile, P15 and P18 experienced marginal decreases in their F1-scores.

**Figure 8:**
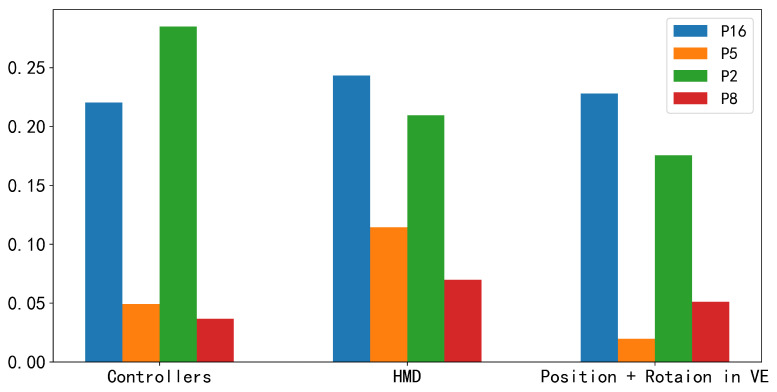
Comparison of each sensor in the personalized model (EEGNet) for those group members who have a limited number of speaking intentions and utterances, quantified by the improvement of F1-score.

We also attempted to only combine different types of leader sensor features with the members’ sensor data to investigate which type of leader sensor feature (HMD, controllers, position, and rotation in VE) contributes the most to the improvement in prediction performance. The position and rotation in the virtual environment represent the leader’s location and orientation. We treat position and rotation as a unified feature for analysis. According to the results shown in Figure 8, for group members P5, P8, and P16, the leader’s HMD sensor data resulted in the most significant improvement (11.5%, 7%, and 24.3%, respectively) in F1-score, while for group member P2, the leader’s controllers sensor data led to the most significant improvement (28.5%) in F1-score. The results indicate that HMD sensor features have a significant impact on enhancing the performance of personalized models for most low-engagement members.

#### 5.3.3. The Qualitative Findings of Members’ Behavior When Holding Intentions to Speak

To further support our previous findings and find some clues about why leader sensor features enhance the prediction of speaking intentions for those low-engagement group members, we attempted to conduct a preliminary analysis of the behaviors of low-engagement group members when they had speaking intentions, focusing on three aspects: hand gestures, head orientation, and body movement. Regarding head orientation, we paid attention to aspects such as the user’s perspective, including where they were looking and at whom. In terms of body movement, we observed the directions and locations of their movements. As for hand gestures, we observed and analyzed the hand gestures of these members referencing the communication gesture types in VR provided by Harrison et al. [54]. Moreover, our analysis and observation primarily relied on watching first-person perspective videos of these low-engagement group members and leaders.

Through our observations and analysis, we found that when examined from the perspectives of hand gestures and body movements, no relevant clues were identified. During the group discussion, these low-engagement group members not only contribute less to expressing their opinions but also exhibit relatively monotonous behavior, mostly moving near the table and whiteboard area. Some individuals (P16, P5) remain stationary for extended periods with no changes in their perspective. There were no particularly distinctive body movements observed when they had speaking intentions. Furthermore, among these low-engagement group members when they had speaking intentions, we did not observe any hand gestures related to the leader. P5, P8, and P16 did not engage in whiteboard ranking during the discussions, and their hand gestures were minimal, with only a few unrelated hand movements, such as adjusting the grip on controllers. P2 participated in ranking two survival items during the discussion and made some hand movements related to holding a pen for writing, but they were unrelated to speaking intentions. From the perspective of head orientation, we found a few clues. Whether it was a successful or unsuccessful speaking intention, we observed that in many cases, these low-engagement group members, when having speaking intentions, would often look towards the leader and other actively discussing group members. Alternatively, they would look at the ranking list on the whiteboard or survival item panel. Furthermore, we observed that while these low-engagement group members were looking towards the leader or other members, the leaders were engaged in discussions with other members or ranking survival items on the whiteboard. Table 6 illustrates the frequency of different head orientation behaviors of these low-engagement group members when they had speaking intentions. Furthermore, we observed that when low-engagement group members held the intention to speak, leaders were almost looking around the survival item panel and whiteboard, engaged in discussions with other high-engagement group members, or organized discussion topics. It seems to be difficult for leaders or other members to recognize being looked at by low-engagement members. Therefore, we speculate that these low-engagement group members, when holding speaking intentions, exhibit the behavior of looking toward the leader or other high-engagement group members, possibly seeking an opportunity to join others’ discussion or expecting assistance from the leader. In such cases, combining the leader’s sensor features may assist the model in better learning the motion patterns of speaking intentions for these low-engagement members.

#### 5.3.4. Suppressed Speaking Intention

In our model training process, we utilized labels for both achieved speaking intentions and unachieved speaking intentions (suppressed speaking intentions). Solely relying on sensor data available on off-the-shelf VR devices, our model found it challenging to distinguish between these two types of speaking intentions. Moreover, through preliminary video analysis, we observed that group members exhibited minimal differences in body movement and gaze when expressing achieved and unachieved speaking intentions. For instance, P16 had numerous unachieved speaking intentions and displayed similar behaviors, whether having achieved or unachieved speaking intentions, such as looking towards the leader and moving around the item panel. Similarly, P6 moved around the item panel and looked at it regardless of the type of speaking intention. Therefore, based solely on motion data, we found it challenging for the model to differentiate between these two distinct speaking intentions. Additionally, the number of unachieved speaking intentions was significantly lower than that of achieved speaking intentions, making it less favorable for building a separate model and conducting a comprehensive analysis of unachieved speaking intentions. For instance, P10 and P4 had very few unachieved speaking intentions. Considering that the members’ behaviors for achieved speaking intentions and suppressed speaking intentions exhibit minimal differences in motion patterns, we believe that using labels for both achieved and unachieved speaking intentions enables the model to comprehensively learn motion patterns related to speaking intentions. Additionally, due to the limited quantity of suppressed speaking intention labels, employing both types of labels concurrently helps alleviate the difficulty in model training.

It is worth noting that although our experiments and methods are conducted in lab settings, our approach could be applied to real-world VR learning environments. With a larger number of participants, model training can leverage data from diverse individuals, indirectly enhancing the model’s generalization ability and providing more accurate results. However, accommodating more participants also implies increased computational resources, thus necessitating more GPU resources for model training. Regarding VR devices themselves, most consumer-grade VR devices feature basic motion sensors (e.g., accelerometers), while some high-end VR devices have a variety of additional sensors (e.g., an eye tracker, biometric sensor, and so forth), allowing our method to adapt to different VR devices. From the user’s perspective, the detection of speaking intention by our method can alleviate issues such as group discussion blockage (e.g., prolonged silence during group discussions). However, detection of speaking intention may also raise privacy concerns (e.g., when users do not wish the leader or other group members to know their intentions to speak). Overall, leaders can utilize information on speaking intention to enhance the efficiency of group discussions and avoid discussion blockages; thus, we believe the benefits can outweigh the drawbacks in relevant situations. In practical development, controlling the permissions of group members to view speaking intention information can minimize users’ privacy concerns.

## 6. Discussion

In this section, we discuss our results and findings and explore the insights these results may offer for assisting leaders in managing group communication and interactions. Additionally, we discuss how our results and findings can provide assistance to developers.

### 6.1. Personalized Model vs. General Model

Our results indicate that using sensor data available on off-the-shelf VR devices can achieve the following results in the general model (EEGNet): Accuracy (0.5823), F1-score (0.6057), and AUROC (0.5633). Furthermore, the performance of the personalized model (EEGNet) is superior to that of the generalization model, with Accuracy (0.6813), F1-score (0.7132), and AUROC (0.6201). Additionally, the lower performance of the general model suggests that there may be significant variations in motion patterns exhibited by individuals when they have speaking intentions. Moreover, the model’s utilization of knowledge learned from a subset of participants is insufficient for detecting the speaking intentions of new users. From an application perspective, if leaders can receive accurately detected speaking intention information, they can better understand the communication needs of group members. However, the lower Precision of the general model results in many incorrect intentions to speak detection results, making it less favorable choice for development. The personalized model outperforms the general model in all evaluation metrics, indicating that, in terms of results, constructing personalized models for each user may be a choice to consider for development. However, building personalized models requires a certain amount of sensor data from group discussions for each user as an initial dataset, which requires more time and effort.

### 6.2. The Influence of Leader’s Contextual Features

On top of the personalized model, we examined the impact of the leader’s sensor features on detecting the speaking intentions of group members. Our results indicate that combining the leader’s sensor features can enhance the detection performance of personalized models for those members who speak less and are less actively involved in discussions. However, the performance of personalized models for actively engaged members does not improve when the leader’s sensor features are combined. Furthermore, we investigated which type of leader’s sensor features (HMD, controllers, position, and rotation in VE) contributes the most to the performance improvement. The results suggest that the HMD sensor features contribute the most to the performance improvement of personalized models for the majority of low-engagement members.

To further support the results obtained through model detection and explore clues to explain the model results, we observed and qualitatively analyzed the behavior of low-engagement group members when they had speaking intentions in experimental videos. We found that in many cases, these low-engagement members would look towards the leader or other actively participating group members when they had speaking intentions. Therefore, we speculate that when these low-engagement members look towards the leader or other high-engagement group members, they may be seeking opportunities to join others’ discussions or expecting assistance from the leader. In this context, the combination of the leader’s sensor features may help the model comprehensively learn the motion patterns of low-engagement members when they hold speaking intentions.

Based on our results and analysis, for low-engagement group members, combining the leader’s sensor features when constructing personalized models can enhance the model’s precision and F1-score. This can help provide more appropriate feedback to the leader, enabling the leader to enhance group communication and obtain more suggestions and ideas for the decision-making process. In the future model construction process, the level of engagement of group members is also a significant interactive feature that requires particular attention. However, before building personalized models, the level of engagement for each group member needs to be assessed in advance. Although participants and leaders could assess the level of engagement to some degree based on informal interactions before group discussions or previous experiences, there are also formal approaches to analyze engagement levels from different perspectives in group discussions or group learning scenarios. For example, behavioral engagement involves sustained on-task behavior during academic activity, including indicators such as persistence, effort, and contributing to the task [52]; social engagement refers to positive socio-emotional interactions; and high-quality social engagement indicates all group members are equally involved in the task [55]. Analyzing the engagement information of group members based on these formal approaches could, however, require a significant amount of additional time and effort.

### 6.3. Insights on Utilizing Speaking Intention Information to Assist the Leader

In this study, we utilized sensor features available on off-the-shelf VR devices to examine the feasibility of detecting leadership opportunities by focusing on the awareness of group members’ suppressed speaking intentions. Speaking intention differs from the feedback used in previous research, such as how often and for how long people talk [56], the number of contributions [57], and the turn of speaking [58]. In addition, from the leader’s perspective, if the leader can be aware of unachieved intentions to speak, they may be able to better balance the participation in group discussions and facilitate more interaction within the group. If the leader can know the speaking intentions of those who may possess limited communication skills or be low-engagement group members, the opportunity could be provided for these members to express their opinions and thoughts. In task-based discussion scenarios, this approach can help leaders encourage more group members to express their views, thereby further enhancing the efficiency of the decision-making process. Moreover, when group discussions encounter obstacles or fall into silence, information about speaking intention from group members can provide a chance for the leader to break the discussion deadlock and make the discussion smooth.

Furthermore, based on our questionnaire results of Q1, there are various reasons for unachieved speaking intentions, such as not wanting to interrupt other members’ discussions and intending to say something that is unrelated to the ongoing discussion. Therefore, providing real-time intervention for every speaking intention may lead to confusion in the discussion. Therefore, it may be advisable for leaders to intervene based on speaking intention information when needed without mistakenly assuming that group members want to express their views immediately. Additionally, when the group discussion encounters difficulties or falls into silence, leaders can access the speaking intention information of group members to explore possible causes and effective interventions.

Our approach can be extended for different types of leadership support in VR-based group discussions by incorporating other detection techniques for groups. For instance, some research indicates a connection between emotion and bodily expression [59,60], suggesting that sensor data from VR can still be used to analyze or detect group members’ emotions to support leaders. Other studies have shown a correlation between turn-taking behavior and non-verbal cues (gaze, head movements, gestures, and body posture) [61].

## 7. Limitation and Future Work

The current study was subject to several limitations. Firstly, there is a limitation regarding generalizability. We attempted to collect data in a problem-based communication scenario (survival game), which may not represent all other discussion activities. The number of participants in our experiment was also relatively small, and they were predominantly young students, which may limit the generalizability of their VR discussion experiences to a broader population. Additionally, variations in individual discussion habits and communication skills could impact participants’ performance in group discussions and might indirectly influence their speaking intentions. Therefore, due to these limitations, we suggest that future research could explore this topic with a more diverse range of discussion contexts and more diverse populations.

Secondly, concerning our annotation method, we employed a retrospective approach for participants to annotate their speaking intentions. This retrospective method may introduce a memory bias problem. We initially considered having participants press buttons on controllers to label their speaking intentions during discussions. However, this approach could potentially increase participants’ cognitive load during discussions as they would constantly need to focus on the task of pressing buttons. The retrospective method circumvented this issue, but post-experiment observations revealed that participants spent a substantial amount of time recalling when they held speaking intentions during the video playback. This could lead to increased participant fatigue and a reduced willingness to engage in thoughtful labeling, and it could potentially compromise the quality of the label data. Therefore, we suggest that future research consider employing additional tools to assist in obtaining more precise and granular label data. For instance, the use of EEG devices or more efficient annotation tools could be applied. For unachieved speaking intention (suppressed speaking intention), relying solely on motion data for model construction and analysis is insufficient. Collecting a sufficient quantity of labels for unachieved speaking intentions is a challenging aspect of detection. Future research could also consider incorporating biometric data for more in-depth analysis and detection of suppressed speaking intention.

Thirdly, in our work, we primarily focused on the sensor features of HMD and the controllers available on VR devices. We also considered participants’ position and rotation information in VE. These features are more readily accessible and do not necessitate additional expertise for model construction. We did not utilize certain biometric features (such as pupil dilation, EEG, and heart rate) or more advanced VR devices with more sensors (such as Meta quest pro and HTC Vive eye pro). Our emphasis was on consumer-level VR devices that are easily accessible to the general population. However, recent research has also utilized biosignals to aid in addressing cognitive-related issues, such as social contexts and emotional autobiographical memory in VR [62,63]. Biometric features may provide additional support for detection and applications. Thus, we acknowledge the potential for these biometric features to enhance the detection of leadership opportunities and suggest that future research explore the integration of these features or other relevant features to improve detection performance and provide better support for leaders.

## 8. Conclusions

In this paper, we examined the feasibility of detecting leadership opportunities in VR group discussions by using sensor data available on off-the-shelf VR devices to detect group members’ speaking intentions. Our approach and results can be used to design the support environment for helping leaders to organize more efficient discussions and foster increased communication among group members. We also investigated whether the sensor features of leaders could enhance the performance of personalized detection models. Our results indicate that, by leveraging sensor features available on off-the-shelf VR devices, the F1-scores achieved on the general model and personalized model were 0.6293 and 0.7132, respectively. These results signify the feasibility of detecting speaking intentions among members of VR-based discussion groups involving a leader, with superior performance obtained in personalized models. We also found that the leader’s sensor features improved the model’s ability for low-engagement group members. After combining the leader’s sensor features, the model’s F1-score, precision, and AUROC could be increased by up to 25.9%, 37.9%, and 9% respectively. To further support our results, we analyzed first-perspective videos to observe the behaviors of low-engagement group members and tried to understand why leader sensor features affect the detection results of low-engagement members. We found that when these members held speaking intentions, they tended to gaze at the leader and other actively participating group members. Our speculation is that they might be seeking opportunities to join discussions with others or anticipating assistance from the leader by using such non-verbal behaviors in leader-led VR-based group discussion settings. Hence, combining the leader’s sensor features might have improved the models’ ability to learn motion patterns associated with the speaking intentions of low-engagement members. Although understanding whether our detection tasks can also achieve similar performance in more discussion scenarios and a border population requires more investigation and validation, our current work can help to advance the technologies for supporting leadership and effective group discussions in a virtual reality-based environment.

## Figures and Tables

**Table 1:** The questions of our post-experiment questionnaire.

Id	Question Content	Question Type
Q1	Have you ever held the intention to speak during the discussion but did not speak out? If yes, please write down the reason.	Open-ended question
Q2	In the group discussion, you were satisfied with the leader’s performance.	5-point Likert scale
Q3	Who do you think contributed the most to the discussion and why?	Open-ended question

**Table 2:** Layer details of our EGG Net model. C is the number of channels (features) in time series data. Legend: “Conv2D”: 2D Convolution Layer, “BN”: Batch-Normalization, “AvgPool”: Average Pooling Layer, “DepConv”: Depthwise Convolution Layer, and “SepConv”: Separable Convolution Layer.

EEG Net Layer		Input Shape
Conv2D	k=(1,11)	C×200
BN		15×C×200
DepConv	k=(C,1)	15×C×200
BN+ELU		30×1×200
AvgPooling	k=(1,4)	30×1×200
Dropout	p=0.25	30×1×50
SepConv	k=(1,16)	30×1×50
BN+ELU		30×1×50
AvgPooling	k=(1,8)	30×1×50
Dropout	p=0.25	30×1×6
Flatten		30×1×6
Dense	$d_{\mathrm{out}}=1$	180

**Table 3:** Performance comparison of personalized model and general model using sensor features (HMD, controllers, position, and rotation in VE). Classification threshold is 0.5. Legend: “Acc”: Accuracy, “Prec.”: Precision, “F1.”: F1-score, and “AUC”: AUROC.

	Personalized Model					General Model				
Models	Acc	F1.	Prec.	Recall	AUC	Acc	F1.	Prec.	Recall	AUC
Baseline	0.5098	0.4889	0.4729	0.5060	0.5071	0.5002	0.4702	0.4522	0.4897	0.4950
EEGNet	**0.6813**	**0.7132**	**0.6201**	0.8393	**0.6058**	**0.5823**	0.6057	**0.5041**	0.7585	**0.5633**
InceptionTime	0.6785	0.7069	0.5908	**0.8798**	0.6053	0.5338	**0.6293**	0.4803	**0.9122**	0.5312
MLSTM-FCN	0.6789	0.6968	0.6084	0.8153	0.5925	0.5383	0.5590	0.4840	0.6616	0.5390

**Table 4:** Comparison of Personalized model (EEGNet) with context and without context of group members who have a limited number of speaking intentions and utterances during VR-based group discussion. The classification threshold is 0.5. Legend: “Acc”: Accuracy, “Prec.”: Precision, “F1.”: F1-score, and “AUC”: AUROC.

	Personalized Model without Context					Personalized Model with Context				
Group Members	Acc	F1.	Prec.	Recall	AUC	Acc	F1.	Prec.	Recall	AUC
P16	0.5172	0.5327	0.3883	0.8481	0.5444	0.7167	0.7914	0.7452	0.8437	0.6290
P5	0.7374	0.6082	0.5184	0.7357	0.6369	0.6955	0.7327	0.6341	0.8676	0.7267
P2	0.4656	0.4928	0.3419	0.8821	0.5190	0.6738	0.7354	0.7213	0.7501	0.5442
P8	0.7256	0.6295	0.4960	0.8614	0.7463	0.6185	0.6801	0.5666	0.8506	0.5751

**Table 5:** Comparison of Personalized model (MLSTM-FCN) with context and without context of group members who have a limited number of speaking intentions and utterances during VR-based group discussion. The classification threshold is 0.5. Legend: “Acc”: Accuracy, “Prec.”: Precision, “F1.”: F1-score, and “AUC”: AUROC.

	Personalized Model without Context					Personalized Model with Context				
Group Members	Acc	F1.	Prec.	Recall	AUC	Acc	F1.	Prec.	Recall	AUC
P16	0.6112	0.4561	0.3766	0.5780	0.6016	0.7648	0.8245	0.7361	0.9371	0.7161
P5	0.7882	0.6570	0.6192	0.6998	0.7298	0.6728	0.7151	0.6047	0.8748	0.6373
P2	0.4467	0.2751	0.1725	0.6786	0.5073	0.7012	0.7731	0.7393	0.8101	0.6134
P8	0.7344	0.4155	0.4197	0.4114	0.6959	0.6288	0.6125	0.5884	0.6387	0.5515

**Table 6:** The frequency of low-engagement members’ head orientation behaviors (where and whom they were looking at) when holding intentions to speak.

Low-Engagement Members	Head Orientation	Count
P2	look around two whiteboards	4
	look at whiteboard and leader	3
	look at the leader and other members	3
	look at whiteboard	2
	look at survival item panel and other members	2
P5	look at survival item panel	12
	look at whiteboard and leader	10
P8	look at whiteboard and survival item panel	7
	look at survival item panel	6
	look at leader and whiteboard	4
	look at whiteboard	2
	look at the leader and other members	1
P16	look at survival item panel and whiteboard	10
	look at leader	7
	look at the leader and other members	6
	look at leader and whiteboard	4
	look at survival item panel	3
	look around two whiteboards	3
	look at whiteboard	2

## Data Availability

The data presented in this study are available upon request from the corresponding author. The data are not publicly available due to privacy reasons.
